# Novel Genetic Variants of GA-Insensitive *Rht-1* Genes in Hexaploid Wheat and Their Potential Agronomic Value

**DOI:** 10.1371/journal.pone.0069690

**Published:** 2013-07-19

**Authors:** Mui-Keng Tan, Jason Koval, Aida Ghalayini

**Affiliations:** 1 Elizabeth Macarthur Agricultural Institute, New South Wales (NSW) Department of Primary Industries, Menangle, New South Wales, Australia; 2 Ramaciotti Centre for Gene Function Analysis, The University of New South Wales, New South Wales, Australia; The Roslin Institute, University of Edinburgh, United Kingdom

## Abstract

This study has found numerous novel genetic variants of GA-insensitive dwarfing genes with potential agricultural value for crop improvement. The cultivar, Spica is a tall genotype and possesses the wild-type genes of *Rht-A1a*, *Rht-B1a* and *Rht-D1a*. The cultivar Quarrion possesses a null mutant in the DELLA motif in each of the 3 genomes. This is a first report of a null mutant of *Rht-A1*. In addition, novel null mutants which differ from reported null alleles of *Rht-B1b, Rht-B1e* and *Rht-D1b* have been found in Quarrion, Carnamah and Whistler. The accession, Aus1408 has an allele of *Rht-B1* with a mutation in the conserved ‘TVHYNP’ N-terminal signal binding domain with possible implications on its sensitivity to GA. Mutations in the conserved C-terminal GRAS domain of *Rht-A1* alleles with possible effects on expression have been found in WW1842, Quarrion and Drysdale. Genetic variants with putative spliceosomal introns in the GRAS domain have been found in all accessions except Spica. Genome-specific cis-sequences about 124 bp upstream of the start codon of the *Rht-1* gene have been identified for each of the three genomes.

## Introduction

Gibberellic acid (GA)-insensitive genes encode mutant proteins that belong to the DELLA subfamily of GRAS regulatory proteins, which are negative regulators of GA responses [1]–[3]. These mutant alleles reduce sensitivity to GA and are orthologues of the *Arabidopsis Gibberellin Insensitive* (*GAI*) gene [1], [4]. These mutant alleles reduce plant height and are thus referred as dwarfing genes. They increase grain yield at the expense of reduced stature [5] and their introduction into cereals had produced high yielding semi-dwarf varieties in an era which had been termed the ‘Green Revolution’ [6].

A number of studies [7]–[9] have reported on the correlation of GA-insensitive *Rht*-*1* alleles with an increase of Hagberg falling number or a decrease in grain α-amylase activity which are important parameters in the industry specification of wheat quality. A recent study [10] to identify QTL for the grain defect, late maturity alpha amylase (LMA) in a doubled haploid wheat population has reported that wheat genotypes with GA-sensitive *Rht-1* alleles have a ‘tall’ phenotype and express severe levels of LMA, whereas most semi-dwarf genotypes with one *Rht-1* dwarfing gene have very low or non-existent LMA expression. The severe dwarfing allele *Rht-B1c* [9] and a 4D locus near the *Rht-D1* gene [11] have also been reported to play a role in seed dormancy. The degree of seed dormancy has a significant impact on the amount of weather damaged kernels from pre-harvest sprouting in the event of rain prior to harvest. These GA insensitive genes have also been reported to be linked to the negative effects of reduced cell elongation resulting in decreased coleoptile length and lower seedling vigour [12].

This group of *Rht-1* genes thus control and/or are associated with physiological processes that not only affect plant height and yield but grain quality and seedling vigour. These genes from wheat were first studied at the molecular level by the sequencing of cDNA clones [13] of the mutant alleles derived from wheat cultivar Norin 10. The original source of these alleles was however derived from a Japanese cultivar, Daruma [6]. Despite the economic and agricultural value of these genes, further characterisation of these genes at the DNA level using conventional sequencing techniques has to date been limited to a few cultivars [14].

Recent advances in sequencing technologies [15]–[16] employ massively parallel approaches to produce millions of short DNA sequence reads in a single run. Wheat has a hexaploid genome and characterization of molecular variation at the gene level is thus more complex than a diploid genome. The capacity for very high throughput sequencing of DNA makes it feasible to study targeted genes in polyploidy genomes on a large scale without the laborious, technically demanding and painstaking task of large scale cloning of gene fragments from the component genomes.

Different machine platforms produce different length reads [16]. The clonal nature of the Roche 454 sequencing system (http://www.454.com/) produces highly accurate sequence reads of 400–500 base pairs in length, suitable for the quantitative discovery of molecular variations including insertions, deletions (indels) and single nucleotide polymorphisms (SNPs). All gene fragments amplified from a target in the genome will be sequenced, giving the pyrosequencing system [17] the invaluable capability for quantitative detection and identification of minor or rare variants. This study utilized the pyrosequencing principle in Roche 454 Sequencing platform to perform a high throughput next-generation deep sequencing of the *Rht-1* gene in seven wheat accessions with the objective to find novel genetic variants that may have value for crop improvement or scientific research. This paper reported on the finding of novel genetic variants of the GA-insensitive *Rht-1* genes in seven wheat accessions and discussed their potential agronomic value.

## Materials and Methods

### Genetic Materials

Seven wheat accessions, obtained from the Australian Winter Cereal Collection, were germinated and extracted for high quality DNA according to published protocol [10]. They were Aus1408, WW1842 (Nudifinia.Inia66/SUN64G), Whistler (Osprey/Hartog//Osprey*2/Kite.sib), Drysdale (Hartog*3/Quarrion), Quarrion, Spica (Three-Seas/Kambourico//Pusa-4/Flora) and Carnamah (RAC-529-911/77-W-660). The wheat line, Aus1408, is a dormant, white-grained wheat from the Transvaal region of South Africa. The accession, Carnamah is a high-yielding semi-dwarf wheat cultivar developed by Dept of Agriculture, WA. The cultivar Spica is an old Australian wheat cultivar with a tall stature and expresses high levels of LMA irrespective of the growing environment [18]. The accessions, WW1842, Whistler and Quarrion are semi-dwarf genotypes bred by the NSW Department of Primary Industries (previously NSW Dept. of Agriculture). Quarrion is an old cultivar with a high ‘transpiration efficiency’ phenotype and was used to backcross into Hartog to give the improved, drought tolerant semi-dwarf cultivar, Drysdale [19], [20].

A total of 180 doubled haploid (DH) lines derived from a cross between WW1842 and Whistler were used to map a genetic variation that has been found in the C-terminal domain of the gene.

### Amplification, Library Formation and Sequencing

Primers (Table 1) were designed from conserved regions of the sequence alignment of published DELLA genes of various Graminae species including *Triticum aestivum* (*Rht-A1a*, *Rht-B1b, Rht-D1b*; Q9ST59), *Oryza sativa* (*SLR1*; BAE96289), *Zea mays* (*D8*; CAB51557), *Sorgum bicolour* (EER93592) and *Hordeum vulgare* (*SLN1;* q8W127). These primers enabled the amplification of seven groups of amplicons that span almost the entire gene from the A, B and D genomes of hexaploid wheat. The locations of the primers were referenced to the GenBank *T. aestivum Rht-B1b* sequence FN649763 (Table 1). The Dell_Rht5’F primer was designed from the alignment of 5′ upstream untranslated region (UTR) regions of the DELLA nuclear transcription factor of *H. vulgare* (*SLN1*, AF460219), *O. sativa* (*GAI*; AC087797; AK242577; NC008396); *Z. mays* (AC190734.2) and *T. aestivum* (AK332917.1). The Dell_Rht5’F primer aligns with a sequence that was conserved across the Graminae species examined and is about 100 to 120 nucleotides (nt) upstream from the start codon of the gene.

**Table 1:** Sequences of the primers used in the amplification of *Rht*-1 gene fragments from hexaploid wheat.

Primer Name	Sequence (5′ to 3′)	nt* position (FN649763)
Dell_Rht5’F	CCAACCCTGGATCCAAATC	2034−2052
Dell_RhtF1	ATGAAGCGSGAGTACCAGGA	2137−2156
Dell_RhtR1	TCTGCGCCACGTCCGCCATGTC	2296−2317
Dell_RhtF2	TCCGACATGGCGGACGTGGC	2295−2312
Dell_RhtR2	GGAGGGCGGGAGATCGAAGTA	2545−2565
Dell_RhtF3	TACTTCGATCTCCCGCCCTC	2545−2564
Dell_RhtR3	CGTGTCSACCACGACGACC	2796−2815
Dell_RhtF4	GTBGTSGTGGTBGACACGCAG	2797−2817
Dell_RhtR4	CTTGATGCCGAAGTCGAC	3154−3171
Dell_RhtF5	GTCGACTTCGGCATCAAG	3154−3171
Dell_RhtR5	GAACATGGTGGAGTAGTAGTG	3604−3624
Dell_RhtF6	CACTACTACTCCACCATGTTC	3604−3624
Dell_RhtR6	CASCCYTCCTTCTCCTCCACC	3915−3935
Rht-F6R6_F1a	GCGGAGCGCACAGAGCGCCA	3769−3788
Rht-F6R6_F1b	GCGCACAGAGCGCCACGAGACT	3776−3797
Rht-F6R6_R1	GCCAGCAGCGTGCTCGCCTGC	3867−3887

* nucleotide.

The 13 primers amplified seven sets of amplicons (5′F/R1; F1/R1; F2/R2; F3/R3; F4/R4; F5/R5 and F6/R6). The PCR profile for the primer pairs (except F3/R3) is an initial denaturation cycle of 95°C for 5 min; 4 cycles of 95°C for 20 s (denaturation), 68°C for 30 s with the annealing temperature decreased by 2°C /cycle to 62°C, 72°C for 30 s (extension); 26 cycles of 95°C for 20 s (denaturation), 62°C for 30 s (annealing), 72°C for 30 s (extension); and a final extension step of 72°C for 5 mins. The thermal cycling parameters for primer pair F3/R3 differed only in the annealing temperature with initial annealing temperature being 62°C, then a 2°C /cycle decrease to 52°C. In order to minimize sequence error reads due to PCR amplification, the Phusion High Fidelity DNA Polymerase (NEB) was used in PCR according to manufacturer’s conditions.

The PCR products were purified by PEG precipitation. This was performed by adding an equal volume of PEG 8000 solution (26.7% PEG 8000 [Promega], 0.6M sodium acetate, 6.5 mM MgCl_2_). The PCR products were precipitated after standing at room temperature for at least 10 min, washed twice with 95% ethanol and air-dried. The purified products were dissolved in TE (pH 8.0) and their concentrations and molecular weight ranges were determined by ethidium bromide staining in comparison with molecular marker standards. The seven sets from one wheat accession were pooled in equi-moles (not equi-concentrations) and with amplicons from another six genes which have been pooled in a similar fashion to form one library of approximately 1.5 µg. Each library was sequenced on the Roche 454 GS FLX platform. Sequencing was performed at the Ramaciotti Centre for Gene Function Analysis, University of New South Wales.

### Data Processing

The sequences in each library were trimmed to remove low quality sequences based on p-value = 0.05 (Table S1). This trim function was performed using the ‘trim’ tool in the CLC Genomics Workbench 6 (www.clcbio.com) with a limit value of 0.05 for the quality trimming and a maximum number of four ambiguous nucleotides at the sequence ends.

The *Rht-B1b* sequence (GenBank FN649763, nt 2034 −4002) of *T. aestivum* was used as the reference sequence to assemble each of the libraries of *Rht-1* gene sequences using the program, SegMan Pro in DNAStar Lasergene 9 (http://www.dnastar.com/). The alignment files (BAM format) are available as a zip file (Material S1).

The program also generated a report listing the SNPs and indels for each accession and their percentage representation with reference to the nucleotide position of the reference sequence. A SNP was called when the percentage of non-reference base at that position (with sequencing depth of at least 40) was at least 20%. Those with lower percentages and/or sequencing depth were cross-checked with the other libraries for corresponding SNPs. They must be present in at least two other libraries (or accessions) with a minimum of 5% in at least one of them to be designated. The combination of the SNP/indel reports of all seven accessions and sorted by the reference positions (RP) of the reference sequence allowed the detection and quantitative comparison of SNPs and indels across the *Rht-1* sequence for the seven accessions (Table S2).

Each representative DNA variant was aligned with homologous regions of the known *Rht-1* genes (see ‘Discussion’) for the identification of new *Rht-1* alleles. Comparison involved both nucleotide and amino acid sequences enabling the detection of new mutations and putative spliceosomal introns (non-coding DNA) in the *Rht-1* genes of accessions examined.

### Mapping of Genetic Variant by High Resolution Melt (HRM)

A rare genetic variant detected in the amplicons from the primer pair; F6/R6 was genotyped using the Type-it HRM PCR kit (Qiagen) according to manufacturer’s instructions. Three primers, Rht-F6R6_F1a, Rht-F6R6_F1b and Rht-F6R6_R1 (Table 1) were used in the HRM assay which was performed in the real-time machine, Rotor-Gene Q (Qiagen). The genotype data were analysed in the program, MapManager QTXb20 [21] to map the genetic locus on the molecular map of WW1842 x Whistler DH population [10].

## Results

The hexaploid wheat accessions comprise six Australian wheat accessions and one South African wheat line namely WW1842, Whistler, Carnamah, Drysdale, Spica, Quarrion and Aus1408. The total number of amplicons sequenced for each of the seven accessions ranged from 7518 to 15805 (Table S1). The raw sequence data of the seven wheat accessions had been submitted to NCBI and their accession numbers are listed in Table S1. The quality and length distributions are consistent across the libraries (Fig. S1). The quality scores based on the Phred scale for the libraries are in the range 20 to 40, suggesting base call accuracies between 99% and 99.99%. This high accuracy was reflected in the outcome of trimming the sequences in each library which left the number of reads after trimming unchanged (Table S1) except for Drysdale where a very small percentage (0.17%) of low quality reads was removed. The trimming resulted in removal of unwanted ambiguous nucleotides at the ends (Table S1).

The amplicons in each *Rht-1* library of an accession comprised a mixture of the wild type GA-sensitive and mutant GA-insensitive dwarfing alleles of the wheat hexaploid genome. The assembly of the sequence reads of the amplicons in each library to the reference sequence, *Rht-B1b* (GenBank FN649763, nt 2034 −4002) enabled the quantitative detection of genetic variations in each library. The clonal nature of pyrosequencing enabled the accurate estimation of the relative frequency representations of the variants and their comparison across accessions (Table S2).

A total of 461 positions of nucleotide polymorphisms comprising 291 SNPs and 159 indels were found across the 7 accessions. As wheat is a hexaploid genome, there were 11 positions that showed both indel and SNP in varying ratios (Table S2). There were 260 SNPs in the coding region, of which 132 did not result in an amino acid substitution in the gene. The numbers of SNPs that resulted in amino acid substitution from 1^st^, 2^nd^ or 3^rd^ position polymorphisms of amino acid codons are 59, 53 and 16 respectively (Table S2).

The coding region of the DELLA gene was divided into 6 sub-regions amplified by 6 pairs of primers (Table 1). The primers were designed from conserved regions of the DELLA genes of Graminae species and thus enabled the amplication of the different alleles from the A, B and D genomes in approximately the relative percent representations in the wheat hexaploid genome.

Each set of sequences amplified by a primer pair comprises varying ratios of variants of the amplicon bounded by that primer pair. An amplicon variant is defined and confirmed by the linkage of a string of both SNPs and indels (Table S2).

### Conserved cis-elements

**Figure 1:**
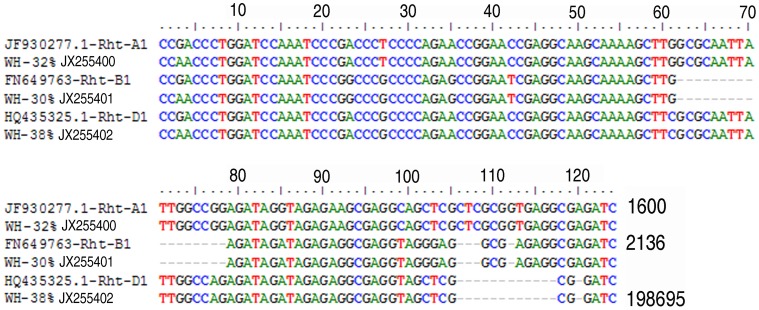
Alignment of 5′ UTR sequences from the cultivar, Whistler (WH) with homologous regions of *Rht-1* alleles in the A, B and D genomes. The percentage denotes the relative proportions of the amplicon from the accession. The RP of the reference sequence, FN649763 is 2034−2136. The GenBank accession numbers for the sequences from Whistler are as denoted.

The pair of primers, Dell_Rht5’F and Dell_RhtR1 amplified a ∼124 bp region upstream of the start codon of the DELLA *Rht-1* gene. Analysis of the 7 libraries found 3 amplicon variants (Fig. 1) amplified in approximately equal ratios. The 3 variants are 124 bp, 103 bp and 94 bp and aligned with 97% identity with homologous 5′ upstream untranslated region (UTR) of the *Rht-A1* (JF930277.1); *Rht-B1* (FN649763) and *Rht-D1* (HQ435324) sequences respectively (Fig. 1).

A total of 44 nt polymorphsims was found in this 124 nt region. There are 28 indels, 14 SNPs and 2 positions with both indel and SNP (Table S2). Except for one SNP, these nt variations define the differentiation of the 3 genome specific cis-elements. The SNP at Reference Position (RP) 41 (Table S2) was found only in Carnamah and is a transition mutation from Adenine (‘A’) to Guanine (‘G’) in the *Rht-B1* allele for ∼10% of the cultivar, Carnamah.

### *Rht-1* Alleles

The primer pair, Dell_RhtF2/Dell_RhtR2 (RP 2293−2565 of FN649763 or RP 260−532 in Table S2) amplified a region that has the two GA signal domains, the DELLA motif and the conserved ‘TVHYNP’ motif of the *Rht-1* gene (GenBank Q9ST59) that are important for binding to the GA receptor, *GID1* [25]–[27].

**Figure 2:**
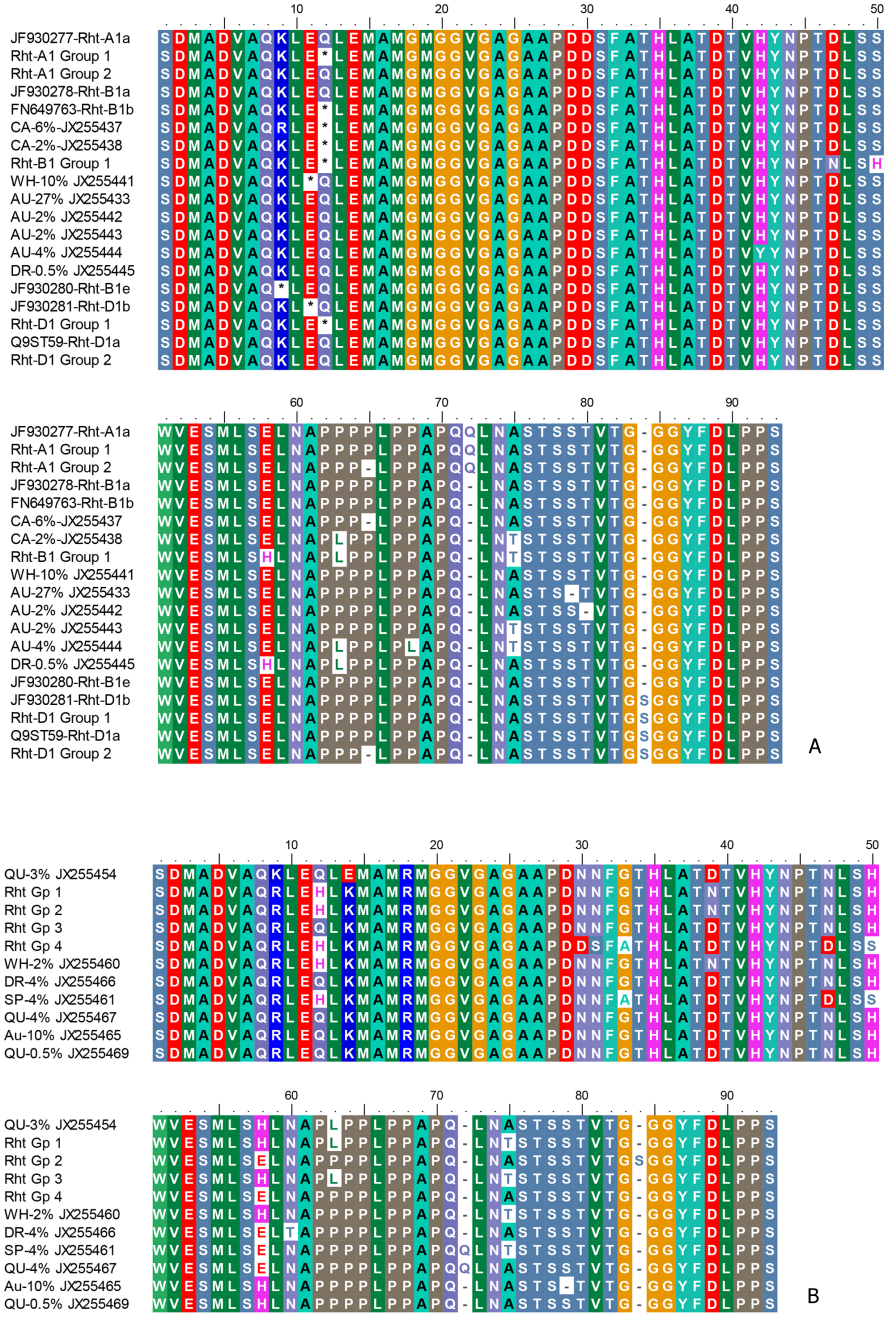
Novel genetic variants at 5′ end of *Rht-1* genes. Alignment of the amino acid sequences translated from DNA sequence amplicons from primer pair, Dell_RhtF2/Dell_RhtR2 (RP 2293−2565 of FN649763 or RP 260−532 in Table S2) of accessions Aus1408 (AU), Whistler (WH), WW1842 (WW), Carnamah (CA), Drysdale (DR), Spica (SP) and Quarrion (QU) with homologous regions of published (**A**) *Rht-A1a*, *Rht-B1*and *Rht-D1* sequences and (**B**) New Variants. Percentage denotes the relative frequency of the amplicon. * denotes a stop codon or an amber mutation. GenBank accession numbers for the sequences are as denoted. Refer to GenBank nt sequences for SNPs that do not result in amino acid substitutions. Amplicon sequences with *Rht-A1a* sequence are AU-37%, WH-23%, WW-42%, DR-33%, CA-42% and SP-40% (GenBank JX255420−JX255425 respectively). Sequences in *Rht-A1* Group 1 include QU-60% (JX255426) and WW-0.5% (JX255427). Sequences in *Rht-A1* Group 2 include WH-18% JX255428) and SP-4% (JX244429). Sequences with *Rht-B1a* include WH-30%, DR-48% and SP-33% (JX255430−JX255432 respectively). Sequences with *Rht-B1b* include WW-34%, QU-26% and CA-7% (JX255434−JX255436 respectively). Sequences in *Rht-B1* Group 1 include QU-2% (JX255440) and CA-0.5% (JX255439). Sequences with *Rht-D1a* include SP-4%, WH-9%, DR-8% and WW-7% (JX255448−JX255451 respectively). Sequences in *Rht-D1* Group 1 include CA-7% (JX255446) and QU-4% (JX255447). Sequences in *Rht-D1* Group 2 include WW-2% (JX255452) and QU-2% (JX255453). Sequences in *Rht* Group 1 include CA-18%, WW-14%, SP-6% and WH-7% (JX255455−JX255458 respectively). Sequences in *Rht* Group 2 include WH-0.1% and SP-1% (JX255459). Sequences in *Rht* Group 3 include AU-17% (JX255464) and DR-2% (JX255468). Sequences in *Rht* Group 4 include CA-4% (JX255462) and SP-1% (JX255463).

Analysis of the sequence data of amplicons from the primer pair, Dell_RhtF2/Dell_RhtR2 differentiated them into groups and comparison with published *Rht-1* sequences enabled the identification of their associated genome (Fig. 2A).

### *Rht-A1*

The major *Rht-A1* allele is *Rht-A1a* and is present in Spica, Whistler, WW1842, Aus1408, Drysdale and Carnamah (Fig. 2A). The cultivar, Quarrion does not possess the wild type *Rht-A1a* gene. The mutant *Rht-A1* allele of Quarrion has a nucleotide transversion of C to T (RP 293, Table S2) which results in a stop codon (Fig. 2A) in the DELLA motif of the gene.

No other stop mutation has been found in the mutant *Rht-A1* allele of Quarrion, it is thus highly likely that this mutant genotype will code for a N-terminal truncated protein as hypothesized by [13]. This is a first report of a mutant allele of *Rht-A1a* with a null mutation. The cultivar, WW1842, has a very minor allele that has the same mutation (Fig. 2A).

Another allele with a deletion of an amino acid, Proline (Fig. 2A), was found in Spica (4%) and Whistler (18%). Thus Whistler and Spica share the same profile of *Rht-A1* gene alleles.

### *Rht-B1*

One of the major amplicon from the primer pair, Dell_RhtF2/Dell_RhtR2, is homologous with the *Rht-B1a* allele and is present in significant proportions of ∼30% in the cultivars; Whistler, Drysdale and Spica (Fig. 2A).

The major variant from WW1842, Quarrion and Carnamah shares a mutation of C to T at RP 293 (Table S2) to result in an amber mutation (Fig. 2A). This is the same position as the stop codon of *Rht-B1b* that gives the semi-dwarf phenotype [13] of these cultivars.

The major *Rht-B1* allele of accession Aus1408 differs from wild type *Rht-B1a* by a Serine deletion (Fig. 2A) corresponding to RP 491−493 (Table S2). It also has 3 other minor *Rht-B1* alleles differentiated by indels and SNPs that result in amino acid substitutions (Fig. 2A). One of the *Rht-B1* allele of Aus1408 has an amino acid substitution of H to Y in the conserved ‘TVHYNP’ signal binding domain (Fig. 2A).

### *Rht-D1*

Sequence analysis showed the presence of the *Rht-D1a* allele in Spica, Whistler, Drysdale, WW1842 and Quarrion (Fig. 2A). In contrast to the occurrence of *Rht-A1a* and *Rht-B1a* alleles in relative proportions of ∼30%, the *Rht-D1a* allele is represented in less than 10% relative frequency in all the accessions analysed (Fig. 2A). A lower copy number will result in a lower transcript level and hence a lower expression level and corroborates with experimental evidence reported in [14].

Whistler has a minor novel allele which has a nucleotide transversion of G to T at RP 290 (Table S2) which results in an amber mutation (Fig. 2A). This stop codon is at the same position as *Rht-D1b* (Fig. 2A).

A mutation at RP 293 (Table S2) that results in a stop codon was observed for two cultivars Carnamah and Quarrion (Fig. 2A). The stop codon is one position downstream of the stop codon of *Rht-D1b*.

### Other Variants

A variant of *Rht-D1a* was not found in Aus1408 (Fig. 2A). Instead, the cultivar, Aus1408 has two alleles that are significantly differentiated from *Rht-D1a* (Fig. 2B). These two Aus1408 alleles (JX255464 and JX255465) are very similar and differ by the indel of one amino acid (Fig. 2B). Variants of these Aus1408 alleles have also been found in all the other accessions (Fig. 2B). The genome assignment of these newly found alleles and their genetic effects remain to be determined.

**Figure 3:**
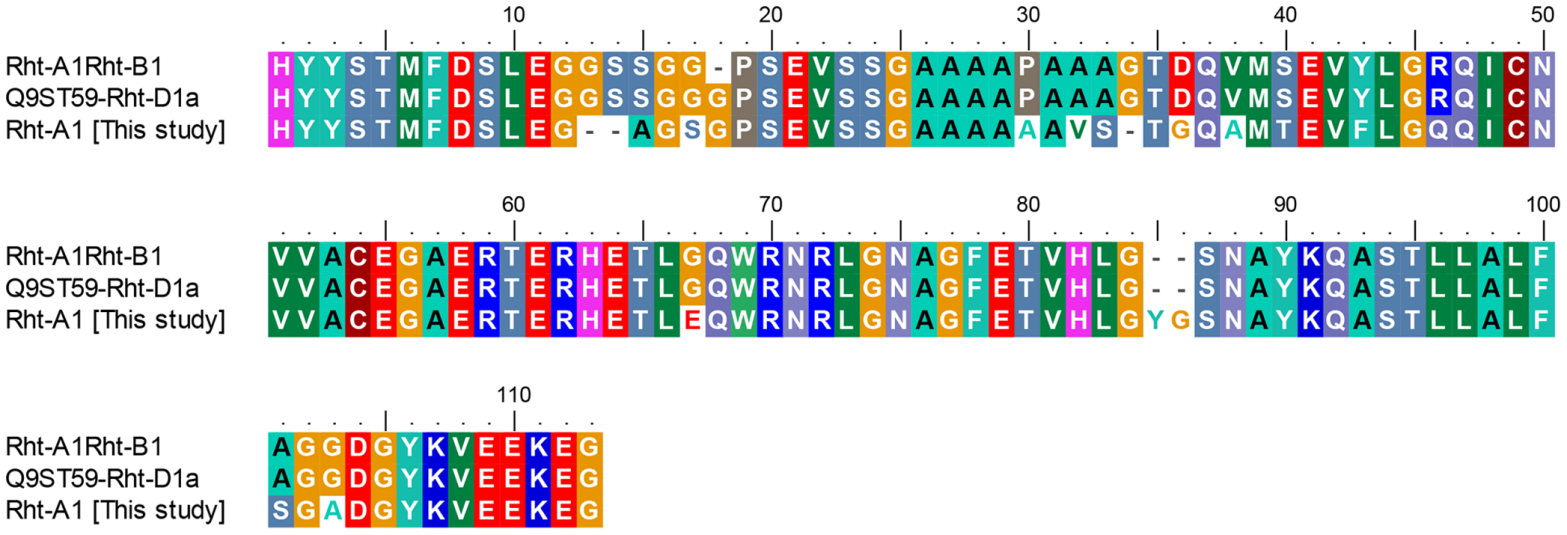
Mutations in the C-terminal GRAS domain of *Rht-A1* alleles. Sequences are translated from DNA sequences of representative amplicons from primer pair, Dell_RhtF6/Dell_RhtR6 (RP 3604−3935 of FN649763 or RP 1571−1902 in Table S2) and aligned. The reference amino acid sequences for *Rht-A1a*/*Rht-B1a* and *Rht-D1a* are Genbank JF930277 (3065−3396) and Q9ST59 (491−601) respectively. Amplicons with sequences similar to *Rht-A1a*/*Rht-B1a* include WH-70%, SP-72%, WW-70%, DR-68%, AU-60%, CA-74% and QU-75% (JX255403−JX255409 respectively). Amplicons with sequences similar to *Rht-D1a* include WH-27%, SP-28%, WW-25%, AU-29%, DR-28%, CA-22% and QU-23% (JX255410−JX255416 respectively). Amplicons with sequences similar to *Rht-A1* include DR-4% (JX255417), WW-5% (JX255418) and QU-3% (JX255419). Please refer to nt sequences in GenBank accession numbers for SNPs that do not result in amino acid substitutions sequences (Table S2).

The region bounded by Dell_RhtF6/Dell_RhtR6 (RP 3604−3935 of FN649763 or RP 1571−1902 in Table S2) is part of the GRAS family transcription factor [30], and is conserved across the published *Rht-A1* and *Rht-B1* sequences and differ from *Rht-D1* only by a glycine insertion (Fig. 3). These 2 groups of conserved amplicons were amplified in the expected ratio of 2∶1 for the 7 accessions (Fig. 3).

**Figure 4:**
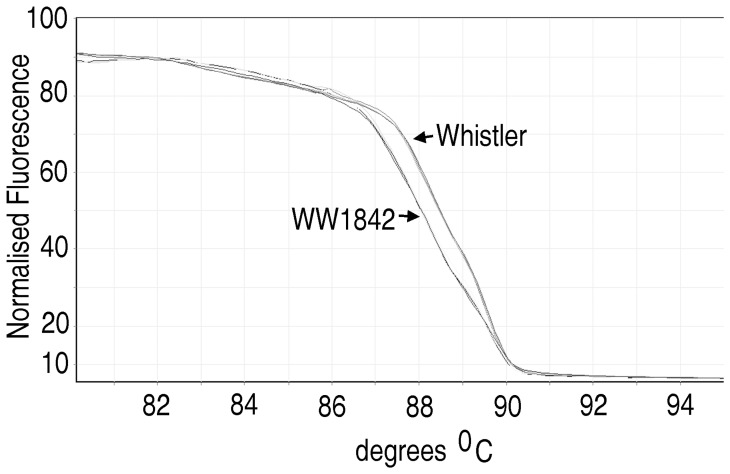
HRM analysis of *Rht-1* variants at the C-terminal GRAS domain in WW1842 X Whistler DH population. Normalized HRM curve for genotyping genetic variation in the *Rht-1* gene bounded by the primers Rht-F6R6_F1a, Rht-F6R6_F1b and Rht-F6R6_FR1 (Table 1). The results are shown for parents, WW1842 and Whistler and eight progeny lines of the DH population (180 lines). Genotype data enabled the mapping of the molecular variation on chromosome 4A.

A distinctive variant characterized by deletions, substitutions and an insertion ‘YG’ and occurring in low relative percentages of 3 to 5% was found for the accessions Drysdale, Quarrion and WW1842 (Fig. 3). The pedigree of Drysdale is Hartog*3/Quarrion and this allele must thus be inherited from Quarrion. The location of this ‘rare’ genetic variation has been determined in this work by mapping this molecular locus on the molecular marker map of WW1842 X Whistler doubled haploid population [10] using HRM (Fig. 4). Mapping the genetic variation involved the design of an allele-specific forward primer for cultivar Whistler (Rht-F6R6_F1a), an allele-specific forward primer for accession, WW1842 (Rht-F6R6_F1b) and a common reverse primer, Rht-F6R6_R1 (Table 1). The two forward primers overlap by 15 nucleotides. All the three primers were incorporated in equi-concentrations in each HRM assay, which gave unequivocal differentiation of the 2 parental alleles in the DH population (Fig. 4). This enabled the high stringency (p = 0.001) mapping of the genetic locus on chromosome 4A at a distance of about 2.4 cM from the DarT marker wPt4424 [10].

### Putative Introns in *Rht-1* genes

The primer pair, Dell_RhtF4/Dell_RhtR4 (RP 2797−3171 of FN649763 or RP 764−1138 in Table S2) amplified a segment of the GRAS family transcription factor [30] that has to date been found to be conserved in the published *Rht-1* genes across the 3 genomes of wheat.

**Figure 5:**
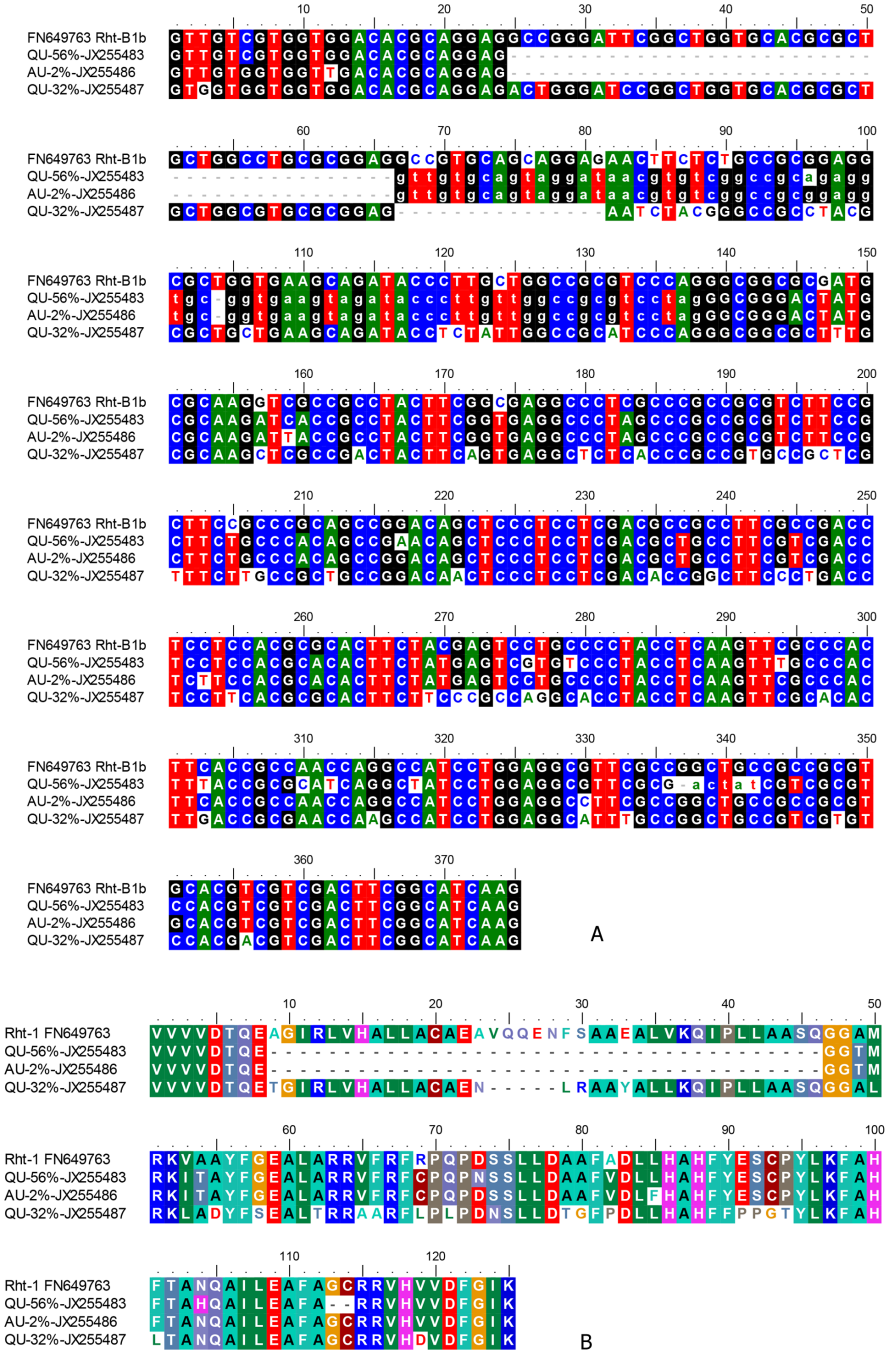
Putative spliceosomal introns in GRAS domain of *Rht-1* genes. Alignment of (A) DNA and (B) amino acid (from translation) sequences of representative amplicons from the primer pair, Dell_RhtF4/Dell_RhtR4 (RP 2797−3171 of FN649763 or RP 764−1138 in Table S2). Bases in lower case are putative intron sequences. Amino acid sequences are translated in Frame 1 from (A). Amplicons with the typical *Rht-1* sequence include SP-100%; WH-97%, WW-95%, DR-88%, AU-68%, CA-50% and QU-12% (JX255470−JX255476 respectively). Amplicons that have the putative intron sequence like QU-56% (JX255483) include WH-2%, WW-1%, DR-6%, AU-12% and CA-45% ((JX255477−JX255482 respectively). Amplicons that have a second intron like AU-2% (JX255486) include WH-0.4% (JX255484) and WW-1% (JX255485). Please refer to nt sequences in GenBank accession numbers for SNPs as annotated in Table S2.

This study found a second group of amplicons from all the accessions except Spica. This second group of amplicons is characterized by a deletion of 42 bases, followed with a nucleotide sequence beginning with ‘GT’ at RP 830 and a base deletion at RP 867 (Fig. 5A; Table S2). A total deletion of 43 bases [(14×3) +1] will shift the reading frame by 2 positions and is indicative of the possible presence of an intron. If the reading frame of the *Rht-1* gene is maintained, this putative intron must extend beyond RP899−901 (TAG) which codes for an amber mutation. An insertion sequence with start ‘GT’ and end with ‘AG’ (Fig. 5A) is typical of a canonical spliceosomal intron [31].

Another base deletion was observed at RP 1099 (Table S2) which again disrupts the reading frame. Due to the presence of 3 closely spaced SNPs at RP 1101, 1104 and 1105 (Table S2), a highly likely scenario is the presence of a 5 bp intron (RP 1099-1104) with the consequent deletion of 2 amino acids ‘GC’ and the resumption of the *Rht-1* coding sequence (Figure 5). The putative intron is bounded by ‘AC’ and ‘AT’ and is typical of a non-canonical spliceosomal intron [31]. The status of the two putative introns remains to be confirmed by experimental evidence. The accessions, Whistler, WW1842 and Aus1408 have additional genetic variants that do not have the deletion at RP1099 and hence do not possess the second putative intron (Fig. 5; Table S2).

The group of shorter amplicons increases in copy number as the ‘type’ sequence decreases in representation (Fig. 5; Table S2). For instance, Spica has 100% of the type sequence and 0% representation of the shorter sequence, Whistler has 97% of the type sequence and 2% of the shorter, variant sequence; Carnamah has approximately equal abundance of the two groups. Quarrion has 12% of the type sequence, 56% of the shorter sequence amplicons and 32% of a third unique variant (Fig. 5). This unique variant has a deletion of 5 amino acids (Fig. 5) and numerous SNPs that result in amino acid substitutions. The start of the deletion coincides with the start of the putative intron sequence (Fig. 5; Table S2).

## Discussion

Deep sequencing using conserved generic primers in the Gramineae family has allowed the detection of numerous genetic variants that have never been detected by conventional cloning and sequencing of these *Rht-1* genes. These genetic variants were identified with high accuracy and specificity for the different wheat accessions. A similar study using pooled multiplexed NGS on a number of genes in a large collection of accessions had reported the detection and quantification of a significant number of rare genetic variants [22]. Previous studies have indicated the reliability and accuracy of finding rare variants in pooled DNA samples using deep sequencing [23], [24].

Genome-specific cis-elements of the *Rht-1* genes have been found to be conserved in the accessions examined except in Carnamah. Carnamah has a minor variant (∼10%) that possesses one unique SNP in the upstream cis-element of the *Rht-B1* allele and the effect of this mutation in this conserved cis-regulating region on the expression of the *Rht-B1* gene of Carnamah would be of interest for further investigation (Fig. 1).

Most of the known mutations in the dwarfing *Rht-1* alleles are null mutations that result in stop codons [14] in the DELLA motif, one of two signal domains of the *Rht-1* genes [25]–[27]. This work presents a first report of a null mutant of *Rht-A1a* in the cultivar Quarrion. The stop codon is at a similar location to the semi-dwarf allele of *Rht-B1b* (Fig. 2A) that confers GA insensitivity. It was postulated in [13] that the null mutants produce N-terminally truncated proteins that reduce or inhibit their binding to the receptors. This results in the reduction of GA-induced degradation of the mutant proteins, which consequently reduces transcriptional responses [28], [29]. It was reported that the *Rht-A1a* allele is expressed at similar levels to the *Rht-B1* homeologs [14]. It will thus be of great agricultural interest to investigate the value of this new null mutant of *Rht-A1* allele (*Rht-A1b*) for crop improvement just like the null mutants; *Rht-B1b* and *Rht-D1b* alleles.

The accessions, Whistler, Drysdale and Spica have the wild type, *Rht-B1a* allele, while the accessions WW1842, Quarrion and Carnamah have the null allele, *Rht-B1b* that gives the semi-dwarf phenotype (Fig. 2A).

The two accessions, Whistler and WW1842 thus have different *Rht-B1* alleles of *Rht-B1a* and *Rht-B1b* respectively. This result confirms the published QTL mapping for LMA in the WW1842 x Whistler doubled haploid position which showed segregation of the *Rht-B1* locus with the *Rht-B1a* allele of Whistler tightly linked or co-located to a highly significant QTL for LMA in the population [10].

Accession Aus1408 does not possess the wild type *Rht-B1a* allele present in Spica or any of the known null mutant alleles. Its *Rht-B1* alleles were characterized by a Serine deletion and an amino acid substitution (Fig. 2A) in the signal binding domain and are predicted to likely alter its binding to the receptor and hence its sensitivity to GA. The value of these alleles for crop improvement is thus worth investigating.

The accessions, Spica, Whistler, Drysdale, WW1842 and Quarrion have the wild type *Rht-D1a* allele (Fig. 2A). A novel null allele of *Rht-D1* was found in Carnamah and Quarrion with the stop codon being one position downstream of the stop codon of *Rht-D1b*. As for *Rht-D1b*, this novel null allele in Carnamah and Quarrion is thus predicted to code for a N-truncated *Rht-D1* product that confers GA insensitivity and reduced plant height and should be of interest for exploitation in breeding.

Whistler has a novel null allele of *Rht-D1* (10%) with the stop codon at the same position as *Rht-D1b* but differs from it by a deletion of a Serine residue (Fig. 2A). This result thus confirms the finding that the WW1842 x Whistler DH population showed segregation at the *Rht-D1* locus with the *Rht-D1a* allele of WW1842 significantly linked to LMA [10]. This also suggests the co-dominance of the two *Rht-D1* alleles (a wild and a null alleles) in Whistler.

Molecular variation characterised by deletions, substitutions and an insertion in the C-terminal conserved regulatory domain of *Rht-1* genes have been found in Drysdale, Quarrion and WW1842 (Fig. 3). This has been mapped to *Rht-A1* on chromosome 4A of the WW1842 x Whistler DH population (Fig. 4). Effects of this molecular variation in this conserved regulatory domain on the expression of the *Rht-A1* allele will have agricultural and scientific interest with potential implications.

Sequence data have provided putative evidence of the existence of a canonical spliceosomal intron of 72 bp and a non-canonical spliceosomal intron of 5bp in the accessions examined except Spica (Fig. 5). The presence of these putative introns also corresponds to deletions of 38 and/or 2 amino acids respectively at the insertion sites (Fig. 5).

The observation on relative frequency representation appears to suggest that the *Rht-1* gene copy numbers in each accession is the same. The increase in the copy number of a genetic variant will be compensated by the decrease in the representation of the wild type allele or another variant. This study has found that the *Rht1* genes in wheat are highly diverse and numerous genetic variants can co-exist in each of the 3 genomes. For instance, Carnamah has three different null alleles of *Rht-B1*, and Whistler has two *Rht-D1* alleles; a wild *Rht-D1a* and a novel, null *Rht-D1* allele (Fig. 2A). The number of permutations of multiple genetic variants in the 3 genomes that can be generated in complex crosses will be huge. The genetic interactions of different combinations and permutations of these variants on expression will need to be studied for their effective utilization in on-going breeding efforts (e.g. [32]) to produce high quality and high yielding wheats.

## Supporting Information

Figure S1 **Quality distributions (Phred scale) (A) and length distributions (B) of amplicons sequenced in each library.**
(TIF)

Table S1 **Total number of reads and their average lengths for each library before and after the ‘trim’ function (limit value = 0.05) of the CLC Genomics Workbench 6 (** www.clcbio.com **).** The NCBI accession numbers for raw sequence data of each library are listed.
(CSV)

Table S2 **Lists of the SNPs and indels in the** ***Rht-1*** **sequences of seven wheat accessions, Spica, Whistler, WW1842, Aus1408, Carnamah, Drysdale and Quarrion with reference to the GenBank FN649763 (** ***Rht-B1b*** **, nt 2034−3935).** The Reference Position (RP) indicated must thus be shifted by 2033 to align on the reference sequence. This table also gives the relative frequencies and sequencing depth of each SNP and indel for each accession. The accessions, Spica, Whistler and Aus1408 have 1944 nt. Cultivar Carnamah has a 3 nt insertion at Contig Position (CP) 712 and so CP of Carnamah with respect to Spica, Whistler and Aus1408 is increased by 3 beyond this point. Cultivars Quarrion, Drysdale and WW1842 have a 6 nt insertion at CP 1859 and so CP of these cultivars with respect to Spica, Whistler and Aus1408 is shifted by 6 beyond this point. The letters under ‘Feature Name/Description’ refer to IUPAC amino acid code. The ‘A’, ‘C’, ‘G’ and ‘T’ in column headings refer to Adenine, Cytosine, Guanine and Thymine respectively.
(XLS)

Material S1 **A zipped folder of separate alignment files (BAM format) of** ***Rht-1*** **gene sequences for wheat accessions; Aus1408, Carnamah, Drysdale, Quarrion, Spica, Whistler and WW1842, which has to be extracted and can be viewed using Tablet (** http://bioinf.scri.ac.uk/tablet **), a second-generation sequencing data visualization tool** [**33**] **.**
(ZIP)
