# Correction: Novel Genetic Variants of GA-Insensitive *Rht-1* Genes in Hexaploid Wheat and Their Potential Agronomic Value

**DOI:** 10.1371/journal.pone.0099375

**Published:** 2014-05-30

**Authors:** The PLOS ONE Staff

After the publication of the article, the reader Peter Chandler raised concerns that several of the new sequences reported are chimeric. In the light of these concerns, we re-evaluated our alignment data and we are issuing this Correction to alert readers that the two novel GA-insensitive mutants of RhtA1 and RhtD1 (JX 255426 and JX255447) reported are chimeric sequences and thus the result of an artefact.

Due to the relatively high rate of chimeric sequences detected for this region, it is envisaged that minor mutants reported in Figure 2 could possibly be a similar artefact of hybrid sequences. Thus all the GenBank sequences submitted for this DELLA region (Figure 2) were examined at the SNP positions that distinguish the three homeologues. This led to the detection of 31 additional GenBank accessions in Figure 2 that are likely to be chimeric sequences. These are JX255427–429; JX255434–435; JX255437–JX255443; JX255445–JX255446; JX255449–455; JX255459–463 and JX255465–469. These sequences have been retracted from GenBank. We are supplying a revised Figure 2 that excludes the chimeric sequences.

**Figure 2:**
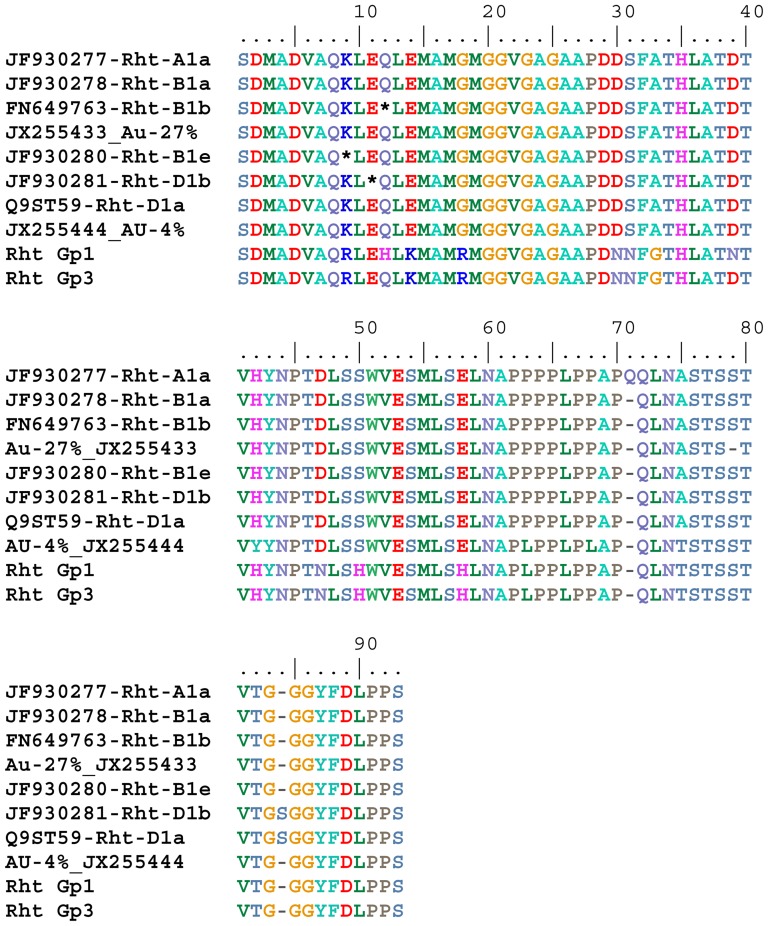
Novel genetic variants at 5' end of Rht-1 genes. Alignment of the amino acid sequences translated from DNA sequence amplicons from primer pair, Dell_RhtF2/Dell_RhtR2 (RP 2293−2565 of FN649763 or RP 260−532 in Table S2) of accessions Aus1408 (AU), Whistler (WH), WW1842 (WW), Carnamah (CA), Drysdale (DR), Spica (SP) and Quarrion (QU) with homologous regions of published Rht-A1a, Rht-B1and Rht-D1 sequences. Percentage denotes the relative frequency of the amplicon. * denotes a stop codon or an amber mutation. GenBank accession numbers for the sequences areas denoted. Amplicon sequences with Rht-A1a sequence are AU-37%, WH-45%, WW-30%, DR-25%, CA-30% and SP-42% (GenBank JX255420−JX255425 respectively) and Quarrion-35%. Sequences with Rht-B1a include WH-38%, DR-55% and SP-47% (JX255430−JX255432 respectively). Sequences with Rht-B1b include WW-27%, QU-33% and CA-11% (JX255436). Sequences with Rht-D1a include SP-5% (JX255448); CA-7%, WW-5% and Quarrion-5%. Sequences with Rht-D1b include WH-3% and DR-1%. Sequences in Rht Group 1 include CA-11%, WW-7% (JX255456), SP-6% (JX255457), and WH-4% (JX255458). Sequences in Rht Group 3 include AU-15% (JX255464) and Quarrion-3%.

The statements made in the article referring to these chimeric sequences as novel mutants are no longer valid. These refer particularly to the GA-insensitive mutant of RhtA1 (retracted JX255426–JX255427) of Quarrion and WW1842 and the GA-insensitive mutant of RhtD1 (retracted JX255446–JX255447) of Quarrion and Carnamah.

The SNP data (See Positions 39, 144, 147 and 150 of Figure 1 in http://www.plosone.org/annotation/listThread.action?root=75721) from the alignment of the sequence JX255444 from Aus1408 suggested the allele may be derived from the D-genome. Hence the RhtD1 allele of Aus1408 is a new variant.

The relative number of sequences representing the A and B genomes in the various libraries is much higher than the relative number of sequences representing the RhtD1 alleles (RhtD1a or RhtD1b) in this region (see corrected Figure 2). There appears to be another allele which is represented as two new variants (corrected Figure 2). This allele has its share of chimeric sequences including Gp 2 and Gp 4 (Fig. 2B) and their GenBank accession numbers have been retracted. The wheat accessions, Carnamah, Spica, WW1842 and Whistler have the Gp 1 variant (corrected Figure 2) and Aus1408 and Quarrion have the Gp 3 variant. Limited SNP data (Position 39 and 150) suggested the new allele may possibly be from the ‘D' genome but this requires further confirmation.

The authors wish to thank Dr. Chandler for raising the issue of chimeric sequences to our attention.
